# The Abuse of Medical Charities

**Published:** 1880-05

**Authors:** 


### THE ABUSE OF MEDICAL CHARITIES.

We recur to this subject again on account of the general interest manifested in it by the profession, both here and abroad, and also on account of the intrinsic importance of the question involved.

It is always much easier to point out evils than it is to remedy them; to find fault with existing institutions than to replace them with better ones.

It is evident to us that those who are just now saying most about the abuses in the medical charities of this city, as well as in New York and elsewhere, have a very inadequate idea of the real causes of these abuses. In nearly all the articles and reports that we have seen on this subject, the medical charities, by which is chiefly meant free dispensaries, are reported as being run in the interest of the medical colleges and most of the abuses are claimed to arise from the efforts of college faculties to increase their clinical material. That we do not misrepresent, the following paragraphs from a recent report will show. The report to which we allude says:

"They (the committee) find, on closer personal investigation, that at least one-third of the patients applying at dispensaries for treatment are in no way entitled to it, and that there is no adequate reason why medicine should be furnished to at least one-half of the remainder.

"They find that the task of attending the sick poor is, by almost common consent, relegated indirectly to the colleges, which are not slow to take advantage of the clinical material thus afforded, and which, having extended this branch of their work beyond all reasonable bounds, are in no small degree responsible for the present deplorable condition of affairs.

"Your committee do not need to study this subject long to see that self-interest is at the bottom of this charitable display and that it matters little to the colleges, *i. e.*, to the individuals composing their faculties, who among the remainder of the profession suffer, so long as they gain by it.

"They find that a somewhat extensive 'ring' controls the administration of medical charity, and that within that 'ring' a few older professors dominate over all the rest. Nor is this the case only in regular circles. It holds just as true among the irregulars. Abundance of evidence convinces them that the trustees and officers of these institutions, both colleges and dispensaries, do not sympathise with any notion to correct the abuses alleged."

We will not stop to comment on the gravity of these plain personal accusations nor upon the modesty of those making them. Neither will we quote similar direct charges against the colleges from the *Medical Record* of New York; but will proceed directly to a brief consideration of the facts; and so far as abuses exist, try to find who is responsible for them and how they are to be remedied. If our memory is not entirely at fault, both the dispensaries in the West and North divisions of this city were organized and maintained for years without the most remote connection with any medical college; indeed when no medical college existed in either of those divisions of the city. They were organized, managed and attended exclusively by the younger class of physicians, who were ambitious to relieve the sick poor, bring themselves into public notice and increase their professional reputation. So far as our knowledge extends, the North Side Dispensary still remains wholly disconnected from any medical college. Since the erection of the new County Hospital in the West Division and the location of the Rush Medical College in the same vicinity, the West Side Central Dispensary has been accommodated with rooms in the basement of the college buildings, but we have failed to see, in the list of names making up the dispensary medical staff, those of any of the older professors; or indeed of any except those of the younger class of medical men, who both attend the patients and give what clinical instruction the institution affords. It is true that the South Side Free Dispensary was organized and sustained for several years by members of the faculty of the Chicago Medical College, but under a legal incorporation entirely distinct from that of the college, and with a corps of attending physicians and surgeons composed almost entirely of the younger members of the profession, two-thirds of whom had no official connection with the college whatever.

With the exception of one or two members belonging to the gynæcological department, the entire dispensary staff is now composed of the younger class of practitioners.

If it is true, as above stated, that the three regular and principal free dispensaries in the city are now, and always have been, served almost exclusively by the younger class of practitioners, a large majority of whom are not members of any college faculty, whose fault is it if one-third of the whole number received and prescribed for were not entitled to such aid? Will it be pretended that the young practitioners serving these several institutions were required by the medical colleges or by any rules of the dispensary boards of trustees, on which some one or two of those "older professors" might have had a place, to examine and prescribe, *nolens volens*, for all who might present themselves whether rich or poor? Or was every member of each dispensary staff at full liberty to exercise his own judgment and promptly turn away any that he might feel sure were able to provide for themselves?

Not only have these attending physicians been thus entirely at liberty to discriminate and restrict their services to the really needy, but the principal founder of the South Side Free Dispensary, who happens to be one of the oldest professors in the city, and who made it a donation, the income from which defrayed a large part of the expenses of the institution for several years, stipulated in writing as the condition on which the donation was made, that the benefits of the dispensary should be *restricted entirely to the actually poor.*

If it is true that one-third of those who have been receiving aid at the several dispensaries are not actually too poor to provide for themselves, then certainly there is a forfeiture of all further claim on the donation alluded to. And yet, who knows but that in the imagination of the young brethren from whose report we have quoted, that same old professor is one of that "extensive ring controlling the administration of medical charity" and even one of the "few who dominate over all the rest." One thing is very certain; and that is, if the trustees of the several dispensaries and charitable medical institutions of this city have erred in the direction of allowing their benefits to be extended to many who were not needy, their error consisted in leaving the matter of discriminating altogether to the members of the attending medical staffs, composed chiefly of young practitioners.

The allegation that the bestowment of indiscriminate medical charity is favored by the faculties of the colleges for the special purpose of increasing their clinical material, is hardly sustained by the facts, so far as relates to the regular schools at least. According to a recent annual report from the trustees of the South Side Free Dispensary, occupying rooms in the basement of the Chicago Medical College, the whole number of visits made by patients during the last year was 11,759; at least three times as many as could be used for any useful clinical purpose by that college.

The number visiting the Central Free Dispensary at the Rush Medical College is still larger. When it is remembered that each of these colleges has ample facilities for clinical instruction in the hospitals with which they are connected, it will be seen that there is certainly no need of their favoring any abuse of medical charity for increasing their clinical material. We have written the foregoing, not from any personal feeling, but simply to show that the outcry against the college faculties, the trustees of dispensaries and the "older professors" was neither founded on facts nor calculated to remedy the abuses complained of. That every medical charity in the city is imposed upon more or less by persons who are able to provide for themselves, we have no doubt. And such will continue to be the case so long as such institutions exist and human nature remains unchanged.

Neither the members of college faculties, nor of the boards of dispensary trustees can stand sentry at the doors to keep the undeserving out. No more can it be expected that the members of the attending staff on duty, will keep up a constant effort at discriminating between the actually needy and those not needy. It is an ungracious kind of work that very few are willing to do day by day. Besides there is no visible basis in dress, good looks, cleanliness, or intelligence, on which one can rely as a mark of distinction between the two classes. To require every applicant to bring a card certifying that he or she is needy, signed by some person presumed to know the circumstances of the applicant, would do some good. But the facility with which such signatures are obtained, and the ease with which the cards can be passed from one person to another, especially under fictitious names, renders this expedient of but limited practical worth. To charge all applicants a small fee, as has been proposed and tried in some quarters, would reduce the number of applicants in a marked degree. But it would reduce the column from the wrong end, by excluding the absolutely destitute who are most needy of all, while those not needy but really able to pay the minimum fees of physicians, would be all the more free to come and avail themselves of the small fee system. The truth is, the abuses so much complained of are inherent in the system of establishing medical charities to which the sick can resort for free medical advice and medicines. And as we stated in a previous number of this journal they cannot be effectually removed except by abandoning the system itself.

And when our younger professional brethren have practically wrestled with the problems involved in every aspect that human ingenuity can devise for thirty or forty years, they will come to the same conclusion. As the report from which we quoted at the commencement of this article, ends with a series of resolutions touching various topics, and which appeared to meet the approval of the society to which the report was read, we append them for the benefit of our readers. They are as follows:

*Resolved*, That this society holds that the trustees or managing officers of dispensaries owe it to themselves and the profession at large to adopt such means as shall most effectually prevent the wholesale abuse of medical charity.

It is understood that this resolution is not intended to militate against the proper administration of medical charity, but against gratuitous services to those not entitled to them.

*Resolved*, That when druggists exceed their proper function as dispensers and chemists, and act in the capacity of prescribers, they usurp a position to which they are not entitled either by experience, knowledge or license, and that this society denounce all such as unworthy of the confidence of the public or the business recommendation of the profession, and advises that its members sedulously avoid any relations with them.

*Resolved*, That this society holds that a physician's prescription does not become public property after passing out of his hands, and should never be either repeated for the same patient or duplicated for another, except by the usual authority of the prescriber.

*Resolved*, We see no justice in treating the clergymen or their families gratuitously, unless they should become objects of charity.

*Resolved*, That the expenses of attending the sick poor, especially at their houses, ought to be borne as all other public expenses are; that physicians should be selected for this work without reference to personal feeling, but simply on the score of ability and character, and that they should be paid, from the public treasury, a sum sufficient to secure adequate and proper service.

---

INFANTILE CONVULSIONS.—(*Archivio Clinico Italiano, No.* 3, 1880.)

Prof. J. Simon, when the children are no longer able to swallow, evacuates the bowels with an abundant simple or oily enema and afterwards injects the following:

| | | | |
|---|---|---|---|
| Musk | (gr. ij) | | 200 |
| Camphor | (℈j) | 1 | |
| Hydrate of chloral | (gr. v) | | 300 |
| Yolk of egg | (℈j) | 1 | |
| Distilled water | (℥v) | 150 | |

